# Effect of Pressure and Particle Size During Aluminum Oxide Air Abrasion on the Flexural Strength of Disperse-Filled Composite and Polymer-Infiltrated Ceramic Network Materials

**DOI:** 10.3390/polym12061396

**Published:** 2020-06-22

**Authors:** Jong-Eun Kim, Jung-Hwa Lim, You-Jung Kang, Jee-Hwan Kim, June-Sung Shim

**Affiliations:** Department of Prosthodontics, Yonsei University College of Dentistry, Yonsei-ro 50-1, Seodaemun-gu, Seoul 03722, Korea; gomyou@yuhs.ac (J.-E.K.); erin850313@gmail.com (J.-H.L.); kyj1219@yuhs.ac (Y.-J.K.); jee917@prostholabs.com (J.-H.K.)

**Keywords:** dental restoration, flexural strength, air abrasion, Weibull analysis, CAD/CAM polymers, disperse-filled composite, polymer-infiltrated ceramic network

## Abstract

Esthetic dental computer-aided design/computer-aided manufacturing (CAD/CAM) polymers such as disperse-filled composites (DFC) and polymer-infiltrated ceramic networks (PICN) should be subjected to surface treatment before bonding. However, such treatment can lead to defect formation and a decrease in strength. Therefore, in this study, we compared the flexural strengths of DFC and PICN materials air-abraded with alumina particles of different sizes at different pressures. In addition to Weibull analysis, the samples (untreated and treated) were characterized by scanning electron microscopy and atomic force microscopy. Both DFC and PICN exhibited the lowest flexural strength at large particle sizes and high pressures. Therefore, we optimized the air abrasion parameters to maintain the flexural strength and significantly increase surface roughness. In the case of DFC, the optimal particle size and pressure conditions were 50 µm at 2 bar and 110 µm at 1 bar, while for PICN, the best performance was obtained using Al_2_O_3_ particles with a size of 50 µm at 1 bar. This study reveals that optimization of the surface treatment process is crucial in the fabrication of high-performance clinical materials for dental restorations.

## 1. Introduction

Computer-aided design/computer-aided manufacturing (CAD/CAM) materials for esthetic restoration that can mimic human tooth color, such as zirconia and glass ceramics, are widely used. Such materials typically require a sintering process that is time-consuming and, in the case of zirconia, results in volume changes [1,2]. Lately, CAD/CAM restorative materials have been produced using ceramics, composite resins, and various fillers. Among them, disperse-filled composites (DFC) and polymer-infiltrated ceramic networks (PICN), which can be differentiated based on their manufacturing method, have attracted considerable interest [3]. The former includes a polymer base mixed with fillers such as zirconia, barium, and silica and is polymerized under standardized industrial conditions, while the latter is produced by infiltration of a polymer into a porous ceramic network [3,4,5,6]. DFC and PICN exhibit improved mechanical properties as they are polymerized at high temperatures and pressures, which makes the entire product homogeneous with a high degree of polymerization [3]. Unlike ordinary ceramics, DFC and PICN materials cause less antagonist wear [7]. Furthermore, production of restorative materials based on DFC and PICN requires a simple milling process without any firing, unlike in the case of glass ceramics or zirconia [8,9].

To attach the intaglio surface of the restoration to a tooth, it should be subjected to surface treatment. Especially at high polymerization rates of the DFC and PICN materials, a lack of appropriate pre-treatment might result in low adhesive strength and high debonding rate [10,11]. Consequently, numerous studies have been conducted on the surface treatment and bonding of polymer-based restorative materials [12,13]. During the bonding of the restorative material, proper surface roughness is essential to achieve adequate mechanical retention. One method to achieve rough surfaces is air abrasion, in which alumina particles are sprayed onto the surface of the material at a constant pressure to produce irregular features. It has been shown that air abrasion with alumina particles is one of the most effective surface treatment methods as it yields rough and clean surfaces, thereby increasing the surface activity of restorative materials [14,15]. Even though the increased surface roughness induced by air abrasion is beneficial for adhesion [12,16], some studies have suggested that surface damage in the form of cracks on the surface of the restorative material may occur during the process, which weakens the restorative material and/or induces mechanical stress [13,17]. Furthermore, severe damage has also been reported on the surfaces of DFC and PICN materials after air abrasion; their adhesiveness could not be improved even after additional surface treatments such as silanization [18,19,20]. A drastic decrease has also been noted in the bonding strength of restorative materials subjected to air abrasion at high pressures (3 bar) [21].

Previous studies on the surface treatment of DFC and PICN materials have compared the results of air abrasion treatment and hydrofluoric acid treatment [22], after air abrasion with particles of a specific size and at a certain pressure [20]. Other studies have involved surface treatment with particles of two different sizes and at different pressure settings, in which only surface roughness and superficial damage were compared qualitatively, and changes in flexural strength were not observed [23]. Evaluation of mechanical properties is crucial for restorative materials such as DFC and PICN that are manufactured and used as dental prostheses in the oral cavity; indeed, ISO standards identify mechanical properties as an important evaluation parameter [24]. Surprisingly, there is not much information or guidelines available for the proper particle size or pressure to be maintained during air abrasion treatment with regard to mechanical strength, which is crucial for clinicians. In this study, we evaluated the effect of air abrasion surface treatment in terms of the flexural strength of DFC and PICN materials subjected to air abrasion at different pressures with alumina of different particle sizes. The null hypothesis of this study is that the flexural strength of DFC and PICN blocks does not change by varying treatment pressure or particle size. 

## 2. Materials and Methods 

Two types of restorative materials, viz., DFC (MAZIC Duro, Vericom Co., Chuncheon, Korea) material containing composite resin with 77 wt. % dispersed nanoparticles and PICN (Vita Enamic, Vita Zahnfabrik, Bad Säckingen, Germany) material containing 86 wt.% reinforced glass ceramic and an infiltrated polymer, were used for testing. To test the flexural strength of surfaces treated by air abrasion and untreated materials, a total of 210 specimens were manufactured and used (fifteen specimens from seven groups of two materials). Blank disks of the DFC (98 mm diameter and 18 mm height) and PICN (98 mm diameter and 30 mm height) materials were cut using a precision cutting machine (ASM100A, Okamoto Co., Tokyo, Japan), trimmed using diamond wheels (#400), and polished with diamond slurries (6, 3, and 1 μm grit). The final dimensions of each specimen were 4 × 1.2 × 18 mm^3^ [24]. Specimen dimensions were measured using a high-precision digital caliper with an accuracy of ±0.001 mm. To maintain certain properties of the materials used in the experiment, the specimens used in this study were produced from one disk per material.

The sample size was determined on the basis of similar studies using dental CAD/CAM polymers [25,26,27,28]. The specimens of each material were divided into seven groups of fifteen samples according to sample size calculation at the 95% confidence level with an alpha value of 0.05 and 80% power; one group of each type was reserved as the control (no surface treatment). The remaining six experimental groups (6 × 15 specimens) were subjected to Al_2_O_3_ air abrasion (Renfert Basic Classic, Renfert GmbH, Hilzingen, Germany) for 10 s with a distance of 10 mm between the air abrasion instrument and target surface [17]. Forty-five specimens were air-abraded using 50 μm Al_2_O_3_ particles (Cobra, Renfert GmbH, Hilzingen, Germany) at 1, 2, and 3 bar (*n* = 15 per pressure group). The remaining 45 specimens were air-abraded with 110 μm Al_2_O_3_ particles (Cobra, Renfert GmbH, Hilzingen, Germany) at 1, 2, and 3 bar (*n* = 15 per pressure group). 

The flexural strength of the specimens (fifteen specimens per group) was determined using a three-point bending test on a universal testing machine (Model 3366, Instron Corporation, Norwood, MA, USA) equipped with a 10 kN load cell at a crosshead speed of 1 mm/min (according to ISO 6872 standards) [24]. Fracture load was recorded in N and the flexural strength (*σ*) was calculated in MPa according to the following relationship:

*σ* = 3*Fl*/2*bh*^2^ (1)

where *F* is the fracture load in N, *l* is the span (distance between the centers of the supporting pins, 14 mm) in mm, *b* is the specimen width in mm, and *h* is the specimen height in mm. 

The Weibull characteristic strength (*σ*_0_) and Weibull modulus (*m*) were calculated according to Equation (2):

(2) $$P_{f}=1-exp\left[-{\left(\frac{\sigma }{\sigma_{0}}\right)}^{m}\right]$$

where *P_f_* is the probability of failure (between zero and one), *σ* is the flexural strength in MPa, and *σ*_0_ is the Weibull characteristic strength in MPa (63.2% of specimen failure) [29].

For morphological analysis, the specimens (4 × 4 × 1.2 mm^3^) were sonically cleaned in distilled water before Pt coating for 60 s (Cressington sputter coater 208HR, Cressington Scientific Instruments, Watford, UK). The specimens were then examined by scanning electron microscopy (SEM, JEOL-7800F, JEOL, Tokyo, Japan) for qualitative (SEM images) analyses. 

The topographical features of the DFC and PICN samples were studied by atomic force microscopy (AFM, NanoWizard 1, JPK Instruments, Berlin, Germany). Owing to size limitations of the AFM device, the specimens were sectioned before evaluation (4 × 4 × 1.2 mm^3^). Two sections were used for each surface treatment group, resulting in a total of 14 specimens for AFM analysis. On each section, nine separate surfaces (5 × 5 μm^2^) were examined and standard descriptors of roughness—arithmetic roughness (*R*_a_), root-mean-square roughness (RMS, *R*_q_), and mean peak-to-valley height (*R*_z_)—were calculated. A cantilever tip (ACTA, Applied Nanostructure Inc., Mountain, CA, USA) with a radius of approximately 2 nm was used in the intermittent contact mode. The scan rate was set at 0.5 Hz and the resonance frequency was in the range of 200–400 kHz. First-order plane-fit image correction was applied on every single record and images were generated at a resolution of 512 × 512 pixels/data point. 

Statistical analysis was performed using SPSS v23.0 (SPSS Inc., Chicago, IL, USA). All obtained data were analyzed for homoscedasticity using Levene’s test and for normal distribution using the Shapiro–Wilk normality test. Two-way ANOVA analysis was performed to determine the influence of air abrasion pressure, Al_2_O_3_ particle size, and their interaction on the mean flexural strength (α < 0.05) of the specimens according to material type. Data corresponding to surface roughness (*R*_a_, *R*_q_, *R*_z_) and flexural strength depending on air abrasion parameters were analyzed by one-way ANOVA followed by post-hoc Bonferroni testing (α < 0.05). The difference in flexural strength between DFC and PICN materials for the same surface treatment method was analyzed by Student’s *t*-test (α < 0.05).

## 3. Results

**Figure 1:**
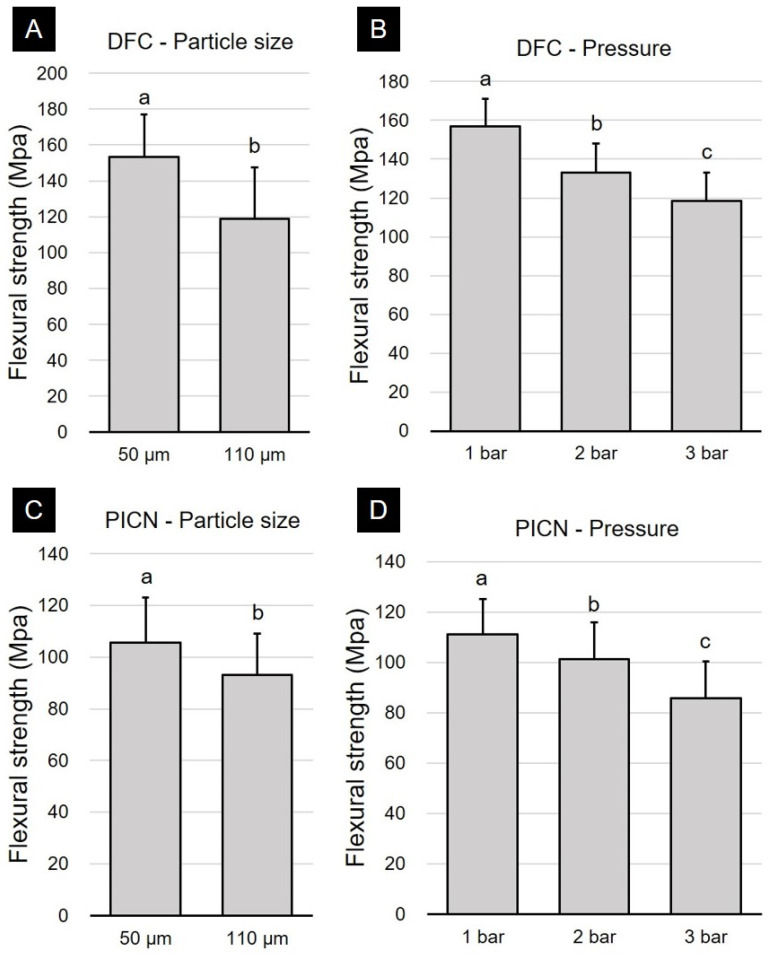
Two-way ANOVA results of surface treatment on the flexural strength of disperse-filled composites (DFC) and polymer-infiltrated ceramic networks (PICN) specimens with respect to (**A**,**C**) Al_2_O_3_ particle size and (**B**,**D**) air abrasion pressure (mean + standard deviation). Different lowercase superscript letters indicate significant differences in flexural strength.

Results for the flexural strength (MPa) and significant differences between the DFC and PICN materials according to surface treatment are presented in Figure 1. Two-way ANOVA of DFC specimens showed that the differences in the particle size (*p* < 0.001) and pressure (*p* < 0.001) significantly affected the flexural strength of the specimen. Significant interactions could be observed between the particle size and pressure (*p* = 0.033). Two-way ANOVA of PICN specimens showed that alumina particle size (*p* < 0.001) and abrasion pressure (*p* < 0.001) significantly affected their flexural strength. However, no significant interactions could be observed between the particle size and pressure (*p* = 0.204). In both materials, it was found that the reduction in flexural strength was significantly greater at a particle size of 110 μm than at 50 μm (Figure 1A,C). In post-hoc testing according to the air abrasion pressure, the reduction in flexural strength was significantly greater at a higher air abrasion pressure (Figure 1B,D). 

**Figure 2:**
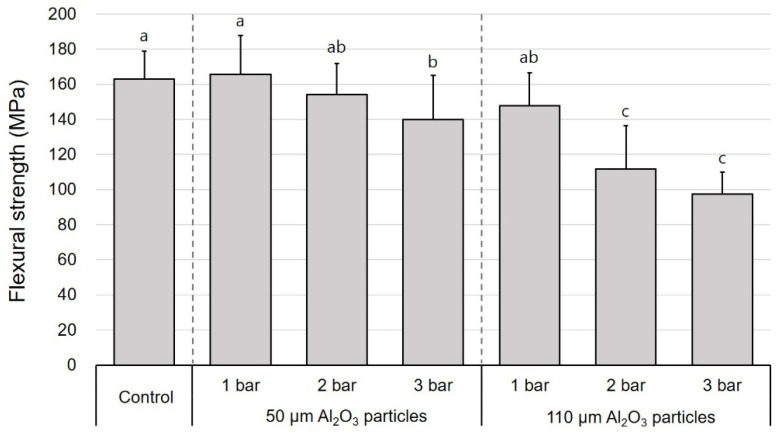
One-way ANOVA and post-hoc test results for the effects of the entire group of surface treatments on the flexural strength of disperse-filled composites specimens with respect to Al_2_O_3_ particle size and air abrasion pressure (mean + standard deviation). Different lowercase superscript letters indicate significant differences in flexural strength.

**Figure 3:**
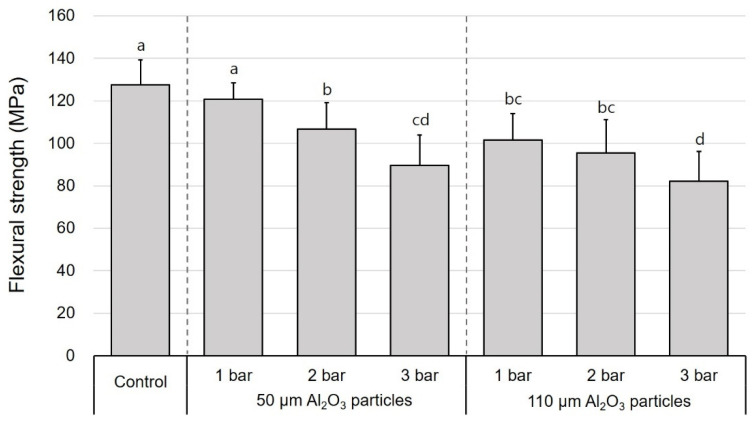
One-way ANOVA and post-hoc test results for the effects of the entire group of surface treatments on the flexural strength of polymer-infiltrated ceramic networks specimens with respect to Al_2_O_3_ particle size and air abrasion pressure (mean + standard deviation). Different lowercase superscript letters indicate significant differences in flexural strength.

**Figure 4:**
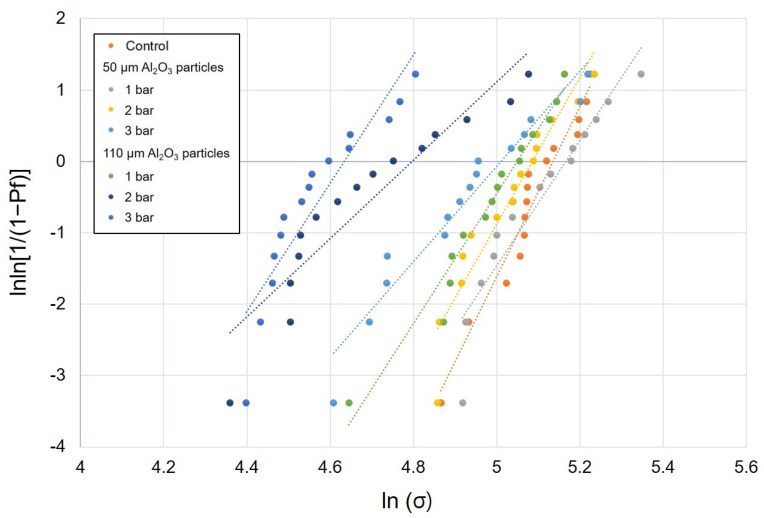
Weibull plot of disperse-filled composites specimens subjected to air abrasion with alumina particles of different sizes at different pressures.

**Figure 5:**
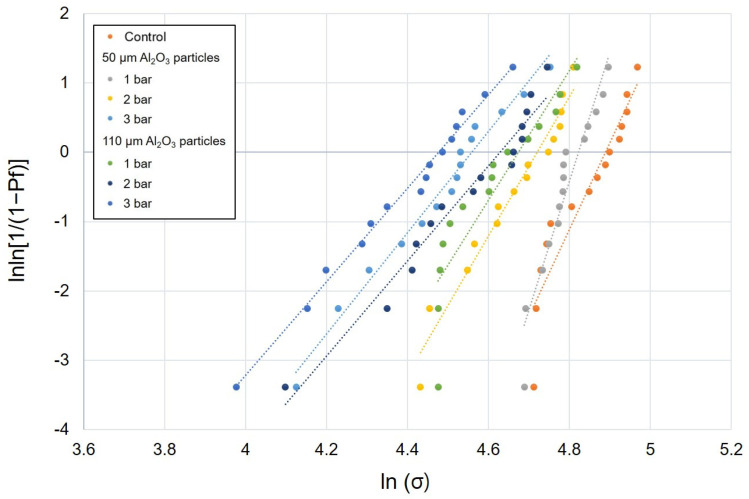
Weibull plot of polymer-infiltrated ceramic networks specimens subjected to air abrasion with alumina particles of different sizes at different pressures.

The mean values of flexural strength, Weibull characteristic strength, and Weibull moduli of the DFC and PICN specimens subjected to air abrasion at different pressures and Al_2_O_3_ particle sizes are presented in Table 1. In all surface treatment groups, there was a significant difference in the flexural strength between the DFC and PICN materials, and the former showed a significantly higher flexural strength. In the case of DFC specimens, the mean flexural strength was in the range of 97.4–165.8 MPa; the lowest value of 97.4 MPa was obtained when surface treatment was carried out with 110 μm sized particles at 3 bar. The second lowest value was obtained with 110 μm sized particles at 2 bar (111.8 MPa). The 50 µm–3 bar group also exhibited a significantly lower flexural strength than the control group (139.9 MPa) (Figure 2). Meanwhile, the mean flexural strength of the PICN samples was in the range of 82.2–121.5 MPa; changes in the flexural strength of both DFC and PICN samples exhibited similar patterns with respect to surface treatments. When air-abraded with 110 μm sized particles, the flexural strength was low at high pressures (2 and 3 bar). However, the 50 μm–3 bar PICN group also showed a low flexural strength, unlike its counterpart DFC group (Figure 3). Similarly, one-way ANOVA and post-hoc analyses confirmed that an increase in pressure and particle size significantly reduced the flexural strength of the specimens. Weibull analysis was performed to measure *σ*_0_ and *m* of each group (Table 1). In both DFC and PICN groups, it was confirmed that the Weibull modulus was high in the control, 50 µm–1 bar, and 50 µm–2 bar groups. In both materials, increasing the particle size or pressure led to a reduction in *m*. The Weibull plots of the DFC and PICN groups are presented in Figure 4 and Figure 5, respectively.

**Figure 6:**
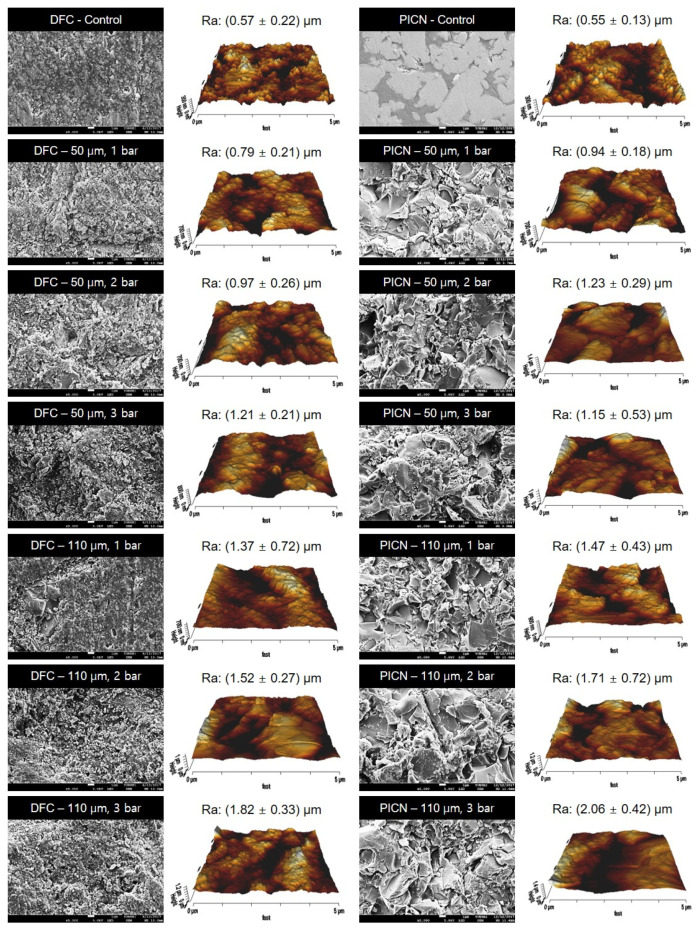
Representative scanning electron microscopy and atomic force microscopy images of different disperse-filled composites (DFC) and polymer-infiltrated ceramic networks (PICN) surface treatment groups.

Representative SEM and AFM images of the different DFC and PICN groups tested are presented in Figure 6. The surface of the untreated DFC control specimen included fillers of different sizes and shapes with a nonuniform and irregular distribution. SEM images of the DFC 50 μm–1 bar treatment group showed that the resin matrix around the fillers was removed by air abrasion, thus exposing the fillers. Increasing the air abrasion pressure and size of the alumina particles resulted not only in the removal of the resin matrix but also in the formation of large defects. More specifically, the SEM images of 50 μm–3 bar, 110 μm–2 bar, and 110 μm–3 bar DFC groups exhibited large defects on the specimen surfaces in addition to cracks. In the SEM images of the PICN specimens, the untreated specimens exhibited a ceramic network structure, which may be attributed to polymer infiltration. Increasing the air abrasion pressure and Al_2_O_3_ particle size increased the surface roughness of the specimens. Sharp edges were formed after surface treatment and increasing the air abrasion pressure resulted in a large number of cracks in the ceramic network and polymer. Several large defects could also be observed. The roughness parameters of each group were analyzed by AFM (Table 2). *R*_a_ and *R*_q_ were positively correlated with alumina particle size and air abrasion pressure. The control groups with no surface treatment exhibited the lowest roughness parameters among all the tested groups. For both DFC and PICN, the 110 μm–3 bar group exhibited the highest roughness.

## 4. Discussion

In this study, to determine the optimal surface treatment conditions for DFC and PICN materials, we evaluated the effect of air abrasion (with alumina of different particle sizes—50 and 110 μm—and pressures—1, 2, and 3 bar) on their flexural strength. The aluminum oxide surface treatment was performed using particles of different sizes and at different pressures in this study because it has been previously reported that appropriate particle sizes or pressures may vary depending on the type of restoration material [20,21,23]. In addition, contextualization of the optimal surface treatment conditions in terms of the flexural strength or surface roughness is quite informative. When 110 μm sized particles were used, as compared to 50 μm sized particles, the surface roughness increased significantly in both DFC and PICN materials on increasing the pressure from 1 to 3 bar; however, it was confirmed that the flexural strength decreased significantly with increasing pressure. Therefore, the null hypothesis of this study can be rejected.

The results of the air abrasion tests at different pressures using alumina particles of different sizes for each material confirmed that the flexural strength of DFC and PICN specimens decreased with increasing alumina particle size and air abrasion pressure. In the case of the DFC specimens, the flexural strengths of the control, 50 µm–1 bar, 50 µm–2 bar, and 110 µm–1 bar groups did not show a statistically significant difference. However, in the case of PICN, except for the 50 µm–1 bar group, all the surface-treated groups showed a significant decrease in the flexural strength when compared to the control group. This observation indicates that PICN is slightly more sensitive to air abrasion than DFC. The lowest flexural strength was observed in the 110 μm–2 bar and 110 μm–3 bar groups for DFC and 110 μm–3 bar and 50 μm–3 bar groups for PICN. 

Studies that evaluate the flexural strength between materials after air abrasion are rare. However, Kurtulmuz-Yilmaz et al. [22] conducted air abrasion at 50 µm–2.8 bar and reported on the flexural strength of Lava Ultimate and Vita Enamic before and after air abrasion. Lava Ultimate is a DFC series material that is produced in a similar manner to the MAZIC Duro used in this study. In Kurtulmuz-Yilmaz’s study, the Lava Ultimate group exhibited a mean flexural strength of 137.2 MPa and it decreased to 120.3 MPa after air abrasion, while the flexural strength of the Vita Enamic group did not vary significantly (116.4 MPa for the control group and 113.9 MPa after air abrasion). This is converse to our findings, as the 50 µm–3 bar group, a similar surface treatment group in our study, showed a significant decrease in flexural strength in both the DFC and PICN groups. In the study by Yoshihara et al. [20], flexural strength was not evaluated, but the surface change after air abrasion was observed, and it was reported that damage to the CAD/CAM polymer surface occurred under 50 µm–2 bar conditions. Lava Ultimate and Cerasmart, produced by combining a disperse filler and a resin matrix, and the PICN-based material Vita Enamic showed the same findings. In our study, the flexural strength decreased significantly in the 50 µm–2 bar group in the PICN group, but the flexural strength did not decrease significantly in the 50 µm–2 bar group for the DFC material, although the roughness increased. 

Similar to the CAD/CAM polymers DFC and PICN evaluated in this study, zirconia requires surface treatment to obtain reliable adhesion [30,31,32,33]. In the zirconia material, several changes in material properties occur on varying the pressure intensity; two contradictory results have been reported on the effect of air abrasion pressure on zirconia material. Some studies reported that surface treatment using air abrasion caused tetragonal–monoclinic transformation toughening and formation of a compressive layer on the surface of the zirconia material that improved its flexural strength [30,31,32]. Other studies have reported that the flexural strength decreased as flaws (e.g., cracks) formed on the surface [33,34]. The DFC material used in our study contains a zirconia filler, but its proportion is too small for the transformation toughening to affect the flexural strength. In addition, because the flaws on the surface, such as cracks to the resin matrix or ceramic network, or loss of the filler, are a more important contributing factor, it is considered that the flexural strength is continuously reduced with increasing particle size or air abrasion pressure.

Weibull analysis also showed that air abrasion treatment negatively affected the mechanical strength and reliability of the DFC and PICN materials. The Weibull modulus represents the structural homogeneity of a material and its strength [29]. In our analysis, in the case of the DFC specimens, the Weibull modulus value changed as the pressure and particle size of the air abrasion increased. There was no tendency to change from the control (untreated) group to the 50 µm–2 bar group, but it decreased in certain groups such as 110 µm–2 bar. However, there was no clear decrease overall. This may mean that after the air abrasion treatment, there is a loss of the resin matrix and the filler is exposed on the surface, such that the results of the surface treatment are inconsistent. In the case of the PICN specimen, the control group showed a Weibull modulus of 12.5, but in the 50 µm–3 bar or 110 µm–3 bar groups, it was found that the value had decreased significantly to 7.3, 6.7, and so on. As the ceramic network of the PICN material has a relatively solid skeleton, it is believed that even by air abrasion treatment, relatively uniform results were obtained depending on the degree of damage to the ceramic network. Previously, it was reported that untreated PICN has a slightly higher Weibull modulus than untreated DFC [35,36]. The Weibull modulus of the untreated specimens presented in our study is consistent with previous studies. However, no published study has evaluated the Weibull modulus after air abrasion surface treatment in DFC and PICN materials. In this study, as the change in the Weibull modulus value is observed after air abrasion surface treatment, the observations are clinically meaningful.

The surface roughness and SEM findings obtained in this study are similar to those of previous studies [20,22,23]. The surface roughness of all the specimens increased with the increasing alumina particle size and abrasion pressure. The SEM analysis results of the specimens that were surface-treated at high pressures with large particles indicated defect formation and filler exposure. Therefore, rather than trying to obtain the highest possible surface roughness, an approach that minimizes damage while obtaining adequate roughness is needed. Several researchers have suggested that surface treatment of DFC and PICN materials by air abrasion should be performed under controlled conditions to prevent damage to the restorative material. Yoshihara et al. analyzed the changes occurring in the adhesive strength and microstructure of DFC and PICN materials when subjected to air abrasion [20]; they found that when surface-treated with 50 µm sized particles at 2 bar, the surface irregularity of both DFC and PICN increased, which increased the adhesive strength of the fabricated restorations. In this case, because silanes can easily access the exposed fillers, the adhesion performance of the restorations was improved [20]. However, cracks formed on the surface and sub-surface of the resin matrix, resulting in a loss of filler particles [20]. Therefore, it may be inferred that the 50 µm–2 bar surface treatment protocol causes significant material damage. An example of one such material is Shofu block HC (Shofu, Kyoto, Japan), whose bonding strength could not be increased even after treatment with silane [20]. Kurtulmus-Yilmaz et al. reported that air abrasion induces an excessive loss of filler particles and damages the resin matrix [22]. Strasser et al. [23] reported similar results on CAD/CAM blocks, with cracked surfaces, loss of filler, and microchipping as the particle size increased or the pressure increased, except for the 50 µm–1 bar and 50 µm–2 bar groups. Therefore, it is imperative to control the surface treatment process to obtain restorative materials with good strength and adhesion performance. 

A limitation of this study is that we conducted the surface treatment operation only for a fixed period of 10 s. More meaningful results may be obtained if the treatment time is optimized as well. In addition, if the cracking mechanism is evaluated using fractography on the specimens of the materials, our understanding of the materials and their properties may be further improved. Another area of future study is adhesion of the DFC and PICN materials to silanes and primers after aluminum-oxide air abrasion using particles of different sizes and at different pressures. Thus, a comprehensive evaluation of how the bonding of these materials to tooth structure or resin cement affects the flexural strength of DFC and PICN restorations after air abrasion treatment would yield clinically more relevant results.

## 5. Conclusions

Within the limitations of the present study, the following conclusions can be drawn. MAZIC Duro (DFC) and Vita Enamic (PICN) exhibited a reduction in flexural strength as the air abrasion aluminum oxide particle size and pressure were increased, although the roughness gradually increased upon surface treatment. The optimal conditions (particle size–pressure) considering the changes in flexural strength and roughness for DFC air abrasion were 50 µm–2 bar or 110 µm–1 bar, whereas for PICN, the optimal conditions were 50 µm–1 bar. To obtain more clinically meaningful results, consideration of adhesive factors is needed through further studies.

Our results imply that the flexural strength of DFC and PICN materials is dependent on both the air pressure during air abrasion treatment and alumina particle size. At inappropriate conditions (high pressures and large particle sizes), filler exposure and resin damage occur. These issues might affect the long-term stability of the prostheses. Therefore, we recommend that, clinically, air abrasion of restorative surfaces should be undertaken with small alumina particles (approximately 50 μm in size) at low pressures.

## Figures and Tables

**Table 1:** Mean and standard deviation of flexural strength and Weibull parameters of DFC and PICN specimens according to air abrasion at different pressures (1, 2, 3 bar) with alumina of different particle sizes (50 µm, 110 µm).

			Flexural Strength (MPa)	*m* (95% CI)	σ_0_ (MPa)
DFC (MAZIC Duro)	Control		162.9 (16.0) ^Aa^	11.9 (10.3–13.6)	169.9
	50 µm	1 bar	165.8 (22.2) ^Aa^	8.8 (7.0–10.6)	175.2
		2 bar	154.1 (17.8) ^ABa^	10.3 (8.4–12.1)	161.7
		3 bar	139.9 (25.0) ^Ba^	6.7 (5.7–7.7)	149.9
	110 µm	1 bar	147.9 (18.6) ^ABa^	9.1 (8.1–10.1)	156.0
		2 bar	111.8 (24.5) ^Ca^	5.5 (4.2–6.7)	121.2
		3 bar	97.4 (12.7) ^Ca^	9.0 (6.6–11.3)	102.9
PICN (Vita Enamic)	Control		127.5 (11.7) ^Ab^	12.5 (9.8–15.2)	132.8
	50 µm	1 bar	120.8 (7.7) ^Ab^	18.6 (15.3–22.0)	124.3
		2 bar	106.7 (12.4) ^Bb^	10.0 (8.8–11.2)	112.1
		3 bar	89.6 (14.4) ^CDb^	7.3 (6.7–7.9)	95.5
	110 µm	1 bar	101.6 (12.4) ^BCb^	9.4 (6.8–11.9)	107.1
		2 bar	95.6 (15.5) ^BCb^	6.9 (6.0–7.7)	102.3
		3 bar	82.2 (14.1) ^Db^	6.7 (6.3–7.1)	88.0

Different uppercase superscript letters indicate significant differences within the column for the assessment of differences in flexural strength depending on the method of surface treatment of the same material. Different lowercase superscript letters indicate significant differences within the column for the assessment of differences in flexural strength between the DFC and PICN materials for the same surface treatment.

**Table 2:** Surface roughness of disperse-filled composites (DFC) and polymer-infiltrated ceramic networks (PICN) groups subjected to air abrasion at different pressures with alumina particles of different sizes.

			*R*_a_ (µm)	*R*_q_ (µm)	*R*_t_ (µm)
DFC (MAZIC Duro)	Control		0.57 ± 0.22 ^a^	0.70 ± 0.26 ^a^	3.10 ± 1.05 ^a^
	50 µm	1 bar	0.79 ± 0.21 ^ab^	0.96 ± 0.25 ^ab^	3.95 ± 0.92 ^ab^
		2 bar	0.97 ± 0.26 ^bc^	1.18 ± 0.28 ^bc^	5.14 ± 1.11 ^bc^
		3 bar	1.21 ± 0.21 ^bcd^	1.45 ± 0.26 ^bcd^	5.78 ± 1.19 ^bc^
	110 µm	1 bar	1.37 ± 0.72 ^cde^	1.70 ± 0.86 ^cde^	5.76 ± 2.31 ^bc^
		2 bar	1.52 ± 0.27 ^de^	1.83 ± 0.32 ^de^	6.74 ± 1.32 ^c^
		3 bar	1.82 ± 0.33 ^e^	2.24 ± 0.30 ^e^	9.62 ± 1.44 ^d^
PICN (Vita Enamic)	Control		0.55 ± 0.13 ^a^	0.65 ± 0.14 ^a^	2.68 ± 0.34 ^a^
	50 µm	1 bar	0.94 ± 0.18 ^b^	1.16 ± 0.20 ^b^	4.81 ± 0.70 ^b^
		2 bar	1.23 ± 0.29 ^bc^	1.48 ± 0.36 ^bc^	5.89 ± 1.06 ^bc^
		3 bar	1.15 ± 0.53 ^bc^	1.49 ± 0.65 ^bc^	6.65 ± 3.00 ^bcd^
	110 µm	1 bar	1.47 ± 0.43 ^bc^	1.78 ± 0.51 ^bcd^	6.59 ± 1.54 ^bcd^
		2 bar	1.71 ± 0.72 ^cd^	2.00 ± 0.79 ^cd^	7.72 ± 2.20 ^cd^
		3 bar	2.06 ± 0.42 ^d^	2.38 ± 0.47 ^d^	8.26 ± 1.41 ^d^

Different lowercase superscript letters indicate significant differences within the column for the assessment of differences in surface roughness depending on the method of surface treatment on the same material. *R*_a_, Roughness average. *R*_q_, Root-mean-square (RMS) roughness. *R*_z_, Average maximum height of the profile.
